# Hepatotoxicity in Rats Induced by Aqueous Extract of Polygoni Multiflori Radix, Root of* Polygonum multiflorum* Related to the Activity Inhibition of CYP1A2 or CYP2E1

**DOI:** 10.1155/2017/9456785

**Published:** 2017-05-24

**Authors:** Deng-Ke Li, Jing Chen, Zhen-Zhen Ge, Zhen-Xiao Sun

**Affiliations:** School of Chinese Materia Medica, Beijing University of Chinese Medicine, 6 Wang Jing Zhong Huan Nan Lu, Chaoyang District, Beijing 100102, China

## Abstract

The objective of this study is to investigate the relationship between the hepatotoxicity induced by Polygoni Multiflori Radix (PMR, root of* Polygonum multiflorum *Thunb., He Shou Wu) and the activity of CYP1A2 or CYP2E1 in the rat liver. Levels of rat serum transaminases ALT and AST were not altered but the activity of CYP1A2 or CYP2E1 in the rat liver was significantly inhibited after oral administration of aqueous extract of PMR under the experimental dosage. However, levels of ALT and AST were significantly increased and the activity of CYP1A2 or CYP2E1 was significantly decreased after injection of specific inhibitor for CYP1A2 or CYP2E1 combined with oral administration of aqueous extract of PMR, especially under the repeated treatment over interval times. Liver histopathological observation showed that a moderate liver injury occurred in rats receiving PMR treatment with the activity of CYP1A2 or CYP2E1 inhibited, but there was no significant liver damage in rats receiving PMR treatment or CYP inhibitor alone. These suggested that low level activity of CYP1A2 or CYP2E1 from genetic polymorphism among people might be one of the important reasons for the hepatotoxicity induced by PMR in clinical practice.

## 1. Introduction

Polygoni Multiflori Radix (PMR, root of* Polygonum multiflorum *Thunb., He Shou Wu) is a traditional Chinese medicine (TCM) that has been used in Chinese clinics for centuries. According to the Pharmacopoeia of the People's Republic of China, the function of PMR is detoxication, eliminating carbuncle, and lubricating the bowels for clinical treatment of sore, carbuncle, scrofulosis, rubella, pruritus, body deficiency in chronic malaria, and constipation [1]. PMR is used widely in China now for different clinical applications such as treating premature graying hair, antiaging, and antihyperlipidemia [2]. Extracts of PMR could completely reverse the C57BL/6 mice hair decolorization induced by H_2_O_2_ with the expressions of *α*-melanocyte-stimulating hormone (*α*-MSH) and melanocortin 1 receptor (MC1R) and tyrosinase (TYP) upregulation [3]. As PMR was proved to inhibit the activity of acetylcholinesterase (AChE) and 2,3,5,4′-tetrahydroxystilbene-2-O-*β*-D-glucopyranoside (THSG) and emodin-8-O-*β*-D-glucoside (EG) as the main components in PMR were proved to decrease AChE activity and THSG increased the expression of protein phosphatase-2A (PP-2A) and microtubule associated protein-2 (MAP-2) in the hippocampus of model rats, PMR was suggested to have the potential for antiaging such as Alzheimer's disease treatment [4, 5]. PMR polysaccharide was reported to have a significant antihyperlipidemic effect in hyperlipidemic mice by its administration at doses of 50 to 200 mg/kg BW for 28 days. The results showed that the serum levels of TC, TG, and AI were significantly decreased, whereas the HDL-C, LPL, HL, and LA levels were significantly increased [6].

Hepatic adverse effects have been frequently reported since PMR is widely used in China and other countries [7, 8]. A metabolomic study on idiosyncratic liver injury induced by different extracts of PMR in rats showed that PMR ethylacetate extract results in evident liver injury, indicated by the significant elevation of plasma alanine aminotransferase and aspartate aminotransferase activities, as well as obvious liver histologic damage. PMR ethylacetate extract had close association with the idiosyncratic hepatotoxicity of PMR and provided a metabolomic insight into idiosyncratic HILI (herb induced liver injury) of different extracts from PMR [9]. RUCAM (Roussel Uclaf Causality Assessment Method) or later also synonymously CIOMS (Council for International Organizations of Medical Sciences) was developed to cope with the shortcomings inherited in the causality assessment of drug induced liver injury (DILI). It is well validated by cases with positive reexposure tests serving as a gold standard. Most importantly, RUCAM is a means of assigning points for clinical, biochemical, and serologic features as well as searching for nondrug causes. There is an update of RUCAM as a development of diagnostic methods and sensitive biomarkers, which made a major step forward to facilitate causality assessment in suspected DILI and HILI cases [10]. One of the systematic reviews on PMR related liver injury reported that a total of 12307 inpatients with liver dysfunction and DILI were screened, which included records of 302 military hospitals in China for the past 10 years. DILI patients, who had taken TCM containing PMR, were assessed by RUCAM scale, showing that 22.5% of the cases are highly probably related to He Shou Wu (RUCAM points > 8), 37.5% of the cases are probably related (RUCAM points 6–8), and 32.5% of the cases are possibly related (RUCAM points 3–5) [11]. Levels of alanine aminotransferase (ALT), aspartate aminotransferase (AST), and alkaline phosphatase (ALP) in serum are the main diagnostic markers for HILI and can be considered the current “golden standard” for initial diagnosis and surveillance [12]. An intrinsic form of HILI such as germander (*Teucrium chamaedrys*) hepatotoxicity is a typical liver injury of the intrinsic form, since it is dose dependent and reproducible in mice [13]. The pathophysiology of idiosyncratic HILI in humans is difficult to assess due to the lack of experimental reproducibility and hence absence of an experimental animal model of HILI. Kava hepatotoxicity is an idiosyncratic liver injury linked to metabolic aberration in unusually susceptible humans, providing an overall low incidence of kava hepatotoxicity in the normal population [14]. We previously reviewed the PMR related acute liver injury in clinical applications in China [15]. It seems that PMR related clinical hepatotoxicity is family related and recurring, suggesting that PMR induced hepatotoxicity might be related to idiosyncratic reaction of patients. Metabolite idiosyncratic DILI is well known to associate with the genetic polymorphisms of liver cytochrome P450 enzyme (CYP450) [16–18].

Polymorphism of* CYP* includes the lack of* CYP* isoforms, loss of inducibility, or synthesis of a CYP form with altered catalytic activity. Polymorphisms have been reported to be related to hepatotoxicity of some drugs in the affected individuals [19, 20]. CYP1A2 and CYP2E1 mainly exist in the liver, accounting for 13% and 7% of total CYP450 enzymes, respectively [21]. In addition, CYP1A2 and CYP2E1 exhibit genetic polymorphism in the population [22, 23]. At least 23 allele genes of CYP1A2 have been found so far, and the major mutants are CYP1A2^*∗*^1C, CYP1A2^*∗*^1D, CYP1A2^*∗*^1F, CYP1A2^*∗*^1K, CYP1A2^*∗*^7, and CYP1A2^*∗*^11. The allele frequency of CYP1A2^*∗*^1C with decreased CYP1A2 activity is approximately 0.22 to 0.25 in the Chinese population [24]. Moreover, CYP1A2 and CYP2E1 are highly conserved among species with 80% identical nucleic acid sequence between humans and rats [25]. The amino acid sequence of CYP1A2 and CYP2E1 in humans and rats has 70% [26] and 80% [27] similarity, respectively. According to a recent study on PMR related hepatotoxicity and genetic polymorphisms of CYP1A2 in clinical patients, the frequency of the CYP1A2^*∗*^1C allele is 46.5% in PMR induced DILI patients, which is significantly different from the frequency of 27.9% observed in healthy people, indicating that the increase of frequency of CYP1A2^*∗*^1C, which decreased the activity of CYP1A2, may be related to the acute liver injury induced by PMR [28]. Recently, we found that enzymatic activity and mRNA expression of CYP1A2 and CYP2E1 in rat liver were significantly inhibited by the aqueous extract of PMR [29], suggesting that the combination of genetic polymorphisms and PMR may induce DILI in rats. This work aimed to investigate whether acute liver injury will occur when PMR is orally administrated to rats with liver CYP1A2 or CYP2E1 inhibited.

## 2. Materials and Methods

### 2.1. Reagents

PMR was purchased from Tong Ren Tang Technologies Co. Ltd. THSG (batch number: 140317), emodin (batch number: 140422), EG (batch number: 140822), and physcion (batch number: 140211) were purchased from Sichuan Weikeqi Biological Technology Co., Ltd. Cimetidine for injection (Jiangsu Shenlong Pharmaceutical Co., Ltd.; standard: 2 mL, 200 mg per ampoule, batch number: 070314) and* trans*-1,2-dichloroethylene were purchased from Sigma Company (product number: C62209-5G).

### 2.2. Preparation of the Aqueous Extract of PMR

PMR was decocted twice in 10x volume of water for 2 hours and 8x volume of water for 1.5 hours, respectively. The extract was merged together and concentrated using rotary evaporators. Then, it was frozen at −80°C and lyophilized using vacuum freezing drying oven. The powder was dissolved in water at a concentration equal to 5.0 g/mL PMR for each experiment. The aqueous extract of PMR is used since water decoction was the most commonly used dosage form for PMR in clinical application.

### 2.3. High Performance Liquid Chromatography (HPLC) Analysis

The HPLC system was a Waters 1525 instrument with a UV detector (Waters). Separation was carried out on a Dikma Diamonsil C18 column (5 *μ*m, 250 × 4.6 mm) at room temperature. A gradient elution program was conducted for chromatographic separation with mobile phase A (0.5% acetic acid) and mobile phase B (acetonitrile) as follows: 0–45 min (10%–35% B), 45–65 min (35%–100% B), and 65–70 min (100% B). The mobile phase was delivered at 1.0 mL/min. The injection volume was 10 *μ*L and the total analysis time was 70 min for each run. The detection wavelength was 280 nm. The powder of PMR and the standard were dissolved with methanol. All tested solutions were filtered through 0.45 *μ*m membrane syringe filters before use. The method followed [30], and the calibration curve was established for the standards: the concentration range of THSG from 46 to 920 *μ*g/mL (*Y* = 29722 × *X* − 125033, *r*^2^ = 0.9998), the concentration range of EG from 1.04 to 41.6 *μ*g/mL (*Y* = 52836 × *X* + 12471, *r*^2^ = 0.9992), the concentration range of emodin from 0.273 to 7.8 *μ*g/mL (*Y* = 66516 × *X* + 9285, *r*^2^ = 0.9996), and the concentration range of emodin methyl ether from 0.111 to 28.86 *μ*g/mL (*Y* = 67576 × *X* − 313, *r*^2^ = 0.9998), and the measurement accuracy and precision are favorable, RSD < 5%.

### 2.4. Animals and Treatment

**Figure 1:**
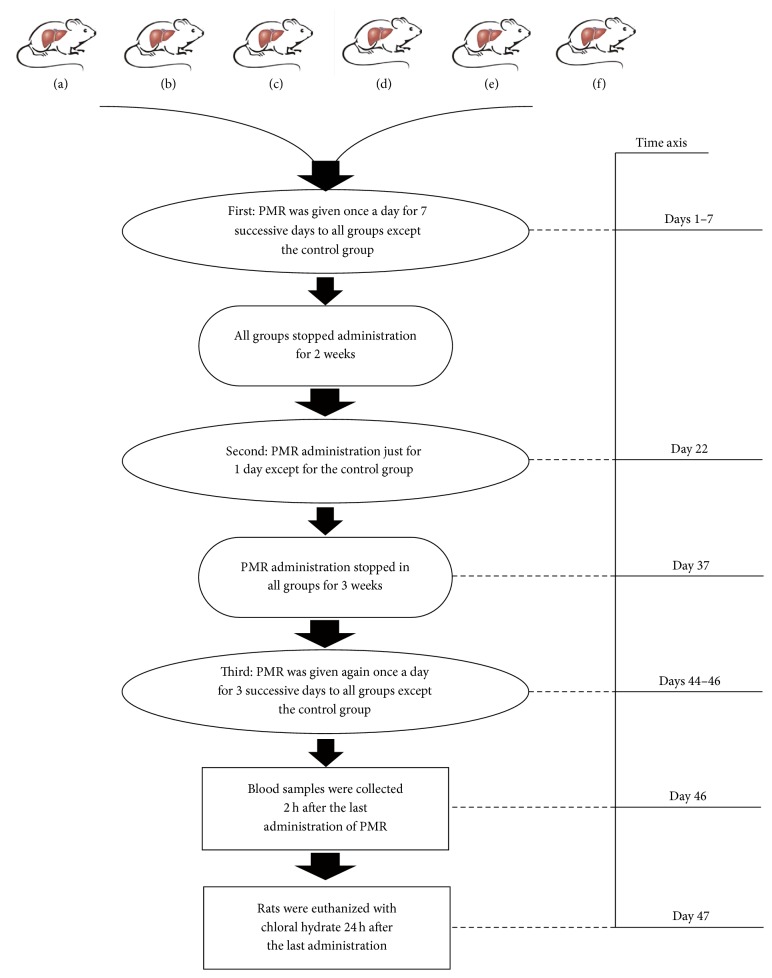
Flow chart of the drug administration. (a) Control group: water only. (b) PMR group: PMR only. (c) CYP1A2 inhibitor control group: cimetidine only. (d) CYP2E1 inhibitor control group:* trans*-1,2-dichloroethylene only. (e) CYP1A2 inhibitor + PMR group: cimetidine and PMR. (f) CYP2E1 inhibitor + PMR group:* trans*-1,2-dichloroethylene and PMR.

Male Sprague-Dawley (SD) rats (200 ± 10 g) were purchased from the Animal Science Center (Peking University Health Science Center, Beijing, China) (production certificate number: SCXK (JING) 2011-0012). The dose of PMR for animal treatment is based on previous animal experiments, which found that receiving an oral administration of PMR at low dose (20 g/kg BW) or high dose (40 g/kg BW) for 60 days did not affect rat serum ALT or AST levels significantly [31]; the effect of CYP450 level was not taken into account in the previous animal experiments. If the level of CYP450 has an effect on PMR related rat liver injury, a high dose may show a more pronounced effect, and the high dose was adopted in this study. Thirty SD rats were randomly divided into six groups (Figure 1): (a) control group, in which the rats received an oral administration of water; (b) PMR group, in which the rats received an oral administration of PMR of 40 g/kg body weight (BW); (c) CYP1A2 inhibitor control group, in which the rats received an intraperitoneal (i.p.) injection of CYP1A2 inhibitor (cimetidine) of 50 mg/kg BW at 5 days prior to the oral administration of water the same as in (a); (d) CYP2E1 inhibitor control group, in which the rats received an i.p. injection of CYP2E1 inhibitor (*trans*-1,2-dichloroethylene) of 100 mg/kg BW at 2.5 h prior to the oral administration of water the same as in (a); (e) CYP1A2 inhibitor and PMR group, in which the rats received an i.p. injection of cimetidine of 50 mg/kg BW at 5 days prior to the oral administration of PMR the same as in (b); (f) CYP2E1 inhibitor and PMR group, in which the rats received an i.p. injection of* trans*-1,2-dichloroethylene of 100 mg/kg BW at 2.5 h prior to the oral administration of PMR the same as in (b). All rats were kept in separate cages under standard conditions (room temperature of 22–27°C, relative humidity of 40%–70%, 12 h light/dark cycle). They had free access to water and a commercial diet before the treatment and during the treatment interval. All animal studies were performed according to the* Guide for the Care and Use of Laboratory Animals* of Beijing University of Chinese Medicine. The animal study protocols were approved by the China Animal Care and Use Committee.

### 2.5. Time Selection to Detect Rat Serum Transaminases in One-Time Treatment of CYP1A2 and CYP2E1 Inhibitor and PMR

According to Section 2.4, rats were i.p. injected with CYP1A2 inhibitor, cimetidine, at 50 mg/kg BW 5 days prior to PMR treatment for (c) and (e) groups and with CYP2E1 inhibitor,* trans*-1,2-dichloroethylene, at 100 mg/kg BW 2.5 h prior to PMR treatment for (d) and (f) groups. Then, the rats in (b), (e), and (f) groups received an oral administration of PM at 40 g/kg BW; besides, (a), (c), and (d) groups received water. The level of serum transaminases ALT and AST of rats was detected at 2 h, 4 h, 24 h, 48 h, and 72 h after the oral administration of PMR.

### 2.6. Oral Administration of PMR to Rats with or without CYPs Inhibited

According to the clinical characteristics of hepatotoxicity induced by PMR [32, 33], we designed an animal experiment with prolonged and repeated oral administration of the aqueous extract of PMR treatment as follows (Figure 1). The animal group setting and treatment of CYP1A2 or CYP2E1 inhibitors were the same as described in Section 2.4. After inhibitor treatment, (b), (e), and (f) groups were fed with PMR at 40 g/kg BW; other groups were fed with water instead, once a day for 1 week. Then, CYP1A2 and CYP2E1 inhibitor treatments and PMR oral administration were all stopped for 2 weeks. Subsequently, rats were treated with CYP1A2 and CYP2E1 inhibitors as above for the second time and then PMR was orally administered just for 1 day. After that, CYP1A2 and CYP2E1 inhibitor treatments and PMR oral administration were stopped again for 3 weeks, and then CYP1A2 and CYP2E1 inhibitors were given; later, PMR was orally administered for 1 to 3 days. Rats were anesthetized with chloral hydrate (0.3 g/kg i.p.) 2 h after the last administration of PMR to examine rat serum ALT and AST levels. Then, rats were euthanized overnight to examine hepatic light microscopic changes and activity of CYP enzymes.

### 2.7. ALT and AST Levels Examination

The serum samples were collected at different times as follows: at day 1, day 3, day 5, and day 7 during the first week of PMR administration, day 22 (the first day after the 2-week interval), day 37 (one day in a 3-week interval), day 44 (the first day after the 3-week interval), and day 46 (the last day of the PMR treatment). The blood samples from rat orbit were collected and centrifuged at 1000 ×g for 10 min, and the supernatant sera were saved. The levels of both ALT and AST were measured using AU-400 fully automatic biochemical analyzer (Olympus Corp.) in the clinical laboratory of Wang Jing Hospital, China Academy of Chinese Medical Sciences.

### 2.8. Pathological Analysis

Briefly, livers of rats from all groups were fixed with 10% formalin and embedded with paraffin. The 5 *μ*m thick sections on the slide were stained with hematoxylin and eosin (HE staining). Pathological examinations of livers were performed by two pathologists using an optical microscope.

### 2.9. Preparation of Liver Microsomes and Measurement of CYPs Activity

Liver microsomes were prepared and the CYP1A2/CYP2E1 activity was measured as described previously [29]. Briefly, 20 *μ*L of drug probes (25 *μ*mol phenacetin and 25 *μ*mol chlorzoxazone) was added to a 5 mL microcentrifuge tube and volatilized to dry. Then, rat liver microsomal protein (2 mg/mL) and NADPH-generation system were added to the tube. The mixture was incubated at 37°C for 30 min and then 600 *μ*L ice-cold acetonitrile was added immediately to stop the reaction. Subsequently, 10 *μ*L of schisandrin (0.06 g/L) was added and the mixture was centrifuged at 10000 ×g for 20 min at 4°C after vortex oscillation. The supernatant was collected from the tube and blown dry with nitrogen, and then 200 *μ*L of (NH_4_)_2_HPO_4_ was added to redissolve. Finally, the solution was centrifuged at 13000 ×g for 15 min and 30 *μ*L of the supernatant was used for HPLC analysis. The percentage for metabolic elimination of each drug probe was calculated using the following equation:

(1) Probe  substrate  metabolic  elimination  percentage=A−BA×100%,

where *A* is the quantity of the added probe substrate and *B* is the quantity of the measured probe substrate.

### 2.10. Statistical Analysis

The data obtained in the assay was expressed in the form of means ± SD. One-factor Analysis of Variance in each group was analyzed with SPSS Statistics 17.0. Statistical significance was set at *P* < 0.05; *P* < 0.01 means extremely significant discrepancy.

## 3. Results

### 3.1. The Main Components in the Aqueous Extract of PMR

**Figure 2:**
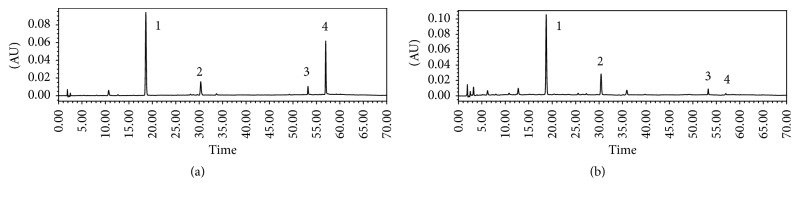
HPLC analysis result of the aqueous extract of PMR. (a) Chromatograms of mixed standard of THSG, EG, emodin, and physcion: 1 for THSG, 2 for EG, 3 for emodin, and 4 for physcion. (b) Chromatogram of the aqueous extract of PMR.

The extraction rate of the aqueous extract was 21.4%. HPLC analysis was adopted to identify the contents of the main components in PMR. In the chromatogram of HPLC, there were four main well-separated chromatographic peaks (Figure 2). They were unambiguously identified as THSG, EG, emodin, and physcion. The components are shown in Table 1.

### 3.2. Treatment with Inhibition of CYP1A2 or CYP2E1 and PMR Administration Significantly Increased Rat ALT and AST Levels at 2 h after One-Time Oral Administration of PMR

**Figure 3:**
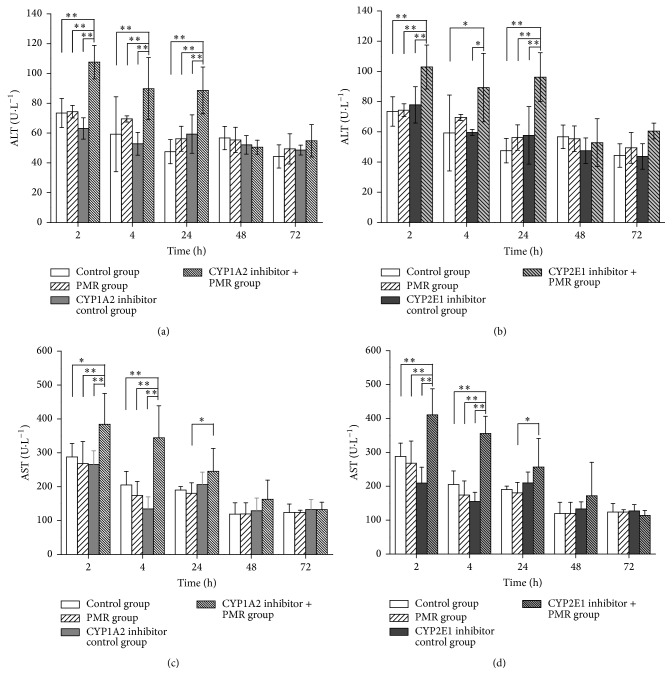
The rat serum transaminases levels assayed at 2 h, 4 h, 24 h, 48 h, and 72 h after oral administration of PMR $\left(\overset{-}{x}\pm s,\;\;n=5\right)$. (a) and (b) for ALT level; (c) and (d) for AST level; (a) and (c) for CYP1A2 inhibited group; (b) and (d) for CYP2E1 inhibited group. ^*∗*^Significant difference compared with other groups, *P* < 0.05. ^*∗∗*^Extremely significant difference compared with other groups, *P* < 0.01.

To determine the optimum time for detecting the serum transaminase level after oral administration of PMR, the ALT and AST levels at 2 h, 4 h, 24 h, 48 h, and 72 h after oral administration of PMR were assayed (Figure 3).

As shown in Figure 3, the administration of PMR along with CYP1A2 inhibitor, cimetidine, or CYP2E1 inhibitor,* trans*-1,2-dichloroethylene, in rats significantly increased the serum transaminases levels within 24 h. Both ALT and AST levels were increased at 2 h after PMR administration (CYP1A2 inhibitor + PMR group compared with control group, PMR group, and CYP1A2 inhibitor control group, *P* < 0.05; CYP2E1 inhibitor + PMR group compared with control group, PMR group, and CYP2E1 inhibitor control group, *P* < 0.05). The AST level kept elevated at 4 h after PMR administration (CYP1A2 inhibitor + PMR group compared with control group, PMR group, and CYP1A2 inhibitor control group, *P* < 0.05; CYP2E1 inhibitor + PMR group compared with control group, PMR group, and CYP2E1 inhibitor control group, *P* < 0.05) and ALT level kept elevated at 24 h after PMR administration (CYP1A2 inhibitor + PMR group compared with control group, PMR group, and CYP1A2 inhibitor control group, *P* < 0.05; CYP2E1 inhibitor + PMR group compared with control group, PMR group, and CYP2E1 inhibitor control group, *P* < 0.05). ALT or AST levels in all groups showed no significant differences after 48 h. As the rat serum ALT and AST levels significantly increased in the experimental groups, which was detected at 2 h after administration of PMR, we choose 2 h after administration of PMR as the time point for detecting the serum transaminase activity in the following experiments.

### 3.3. Rat Serum ALT and AST Levels Significantly Increased after Prolonged and Repeated Administration of PMR

**Figure 4:**
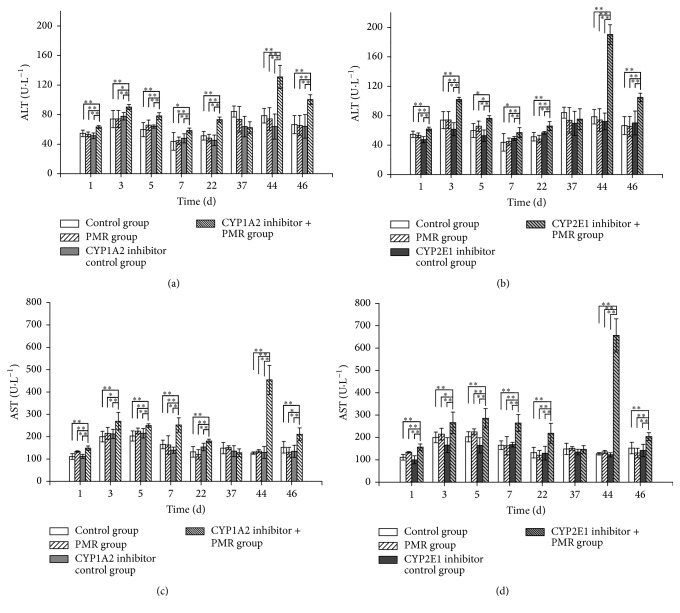
The measurements of transaminases levels at 1 d, 3 d, 5 d, 7 d, 22 d, 44 d, and 46 d after prolonged and repeated oral administration of PMR: oral administration of PMR to rats for 1–7 d, 1 d after 2 w stop (interval), 1–3 d for 3 w stop, and 37 d for transaminases level during oral administration interval $\left(\overset{-}{x}\pm s,\;\;n=5\right)$. (a) and (b) for ALT level. (c) and (d) for AST level. ^*∗*^Significant difference compared with other groups, *P* < 0.05. ^*∗∗*^Extremely significant difference compared with other groups, *P* < 0.01.

Rat serum ALT and AST levels in all groups were assayed at 2 h after administration of PMR as described in Section 2.6. As shown in Figure 4, rat serum ALT and AST levels significantly increased when CYP1A2 or CYP2E1 activities were inhibited before PMR administration (*P* < 0.01). Rat serum ALT and AST levels significantly increased in combination group with administration of PMR and CYP1A2 or CYP2E1 inhibitor compared with all control groups at 1 d, 3 d, 5 d, 7 d, and 22 d (*P* < 0.05), whereas PMR control groups and CYP inhibitor control groups showed no significant increase, which indicated that PMR could induce liver injury in CYP1A2 or CYP2E1 inhibited rats. In order to eliminate false positives, ALT and AST levels among all groups in the interval days (at 37 d from the experiment start, e.g., in the middle of 3-week interval days, Figure 1) were tested, which showed no significant group difference. After the 3-week interval, the rats were treated with PMR again and then serum ALT and AST were assayed; ALT and AST levels extremely significantly increased (*P* < 0.01) at 44 d and 46 d, whereas ALT and AST levels increased nearly twofold compared to all control groups at 44 d which showed more severe liver damage.

### 3.4. Moderate Liver Injury Occurred in Rats after Administration of PMR and Inhibitor of CYP1A2 or CYP2E1

**Figure 5:**
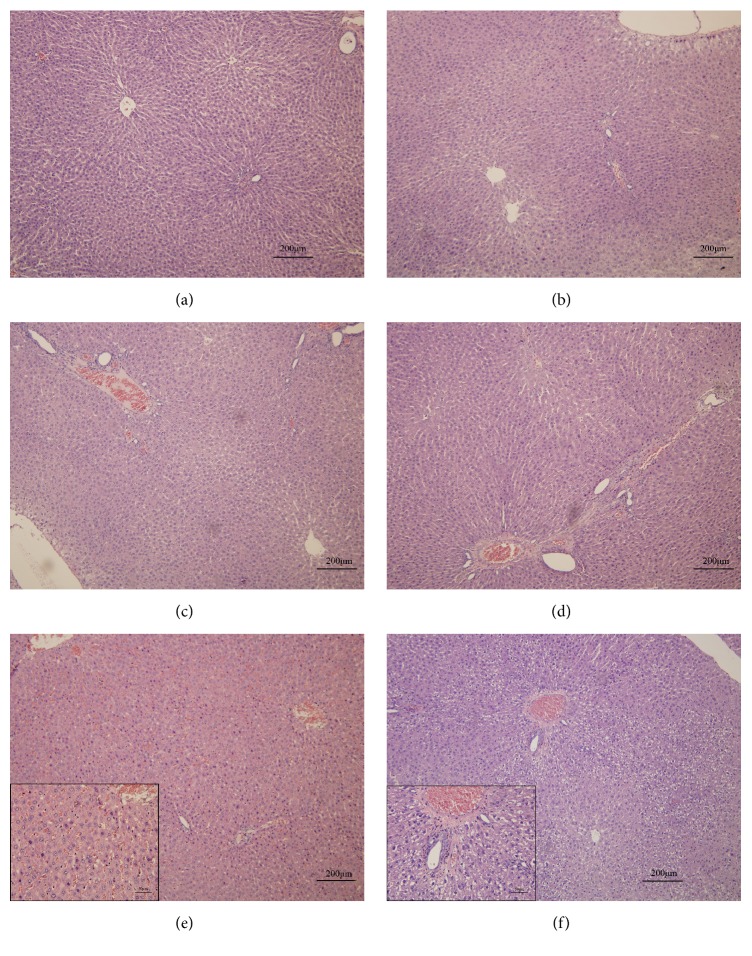
Typical histopathological section photographs of rat hepatic light microscopic changes after administration of PMR and inhibitor of CYP1A2 or CYP2E1. Sections of rat livers from all groups were stained with HE and examined under light microscope (Olympus BX51). (a) Control group; (b) PMR group; (c) CYP1A2 inhibitor control group; (d) CYP2E1 inhibitor control group; (e) CYP1A2 inhibitor and PMR group; (f) CYP2E1 inhibitor and PMR group. The enlarged panels in (e) and (f) indicate the details of pathologic changes.

Rat liver pathological observation is shown in Figure 5; compared with the control group (a), PMR group (b), CYP1A2 inhibitor control group (c), and CYP2E1 inhibitor group (d), CYP1A2 inhibitor + PMR group (e) showed hepatic sinusoid moderate expansion and congestion in the portal tract with some cell karyopyknosis that is an early stage of apoptosis, while CYP2E1 inhibitor + PMR group (f) showed cytoplasmic hydropic change in hepatocytes and hepatic sinusoid moderate expansion in the portal tract.

### 3.5. The Activity of CYP1A2 and CYP2E1 in Rat Liver after Prolonged and Repeated Oral Administration of PMR

**Figure 6:**
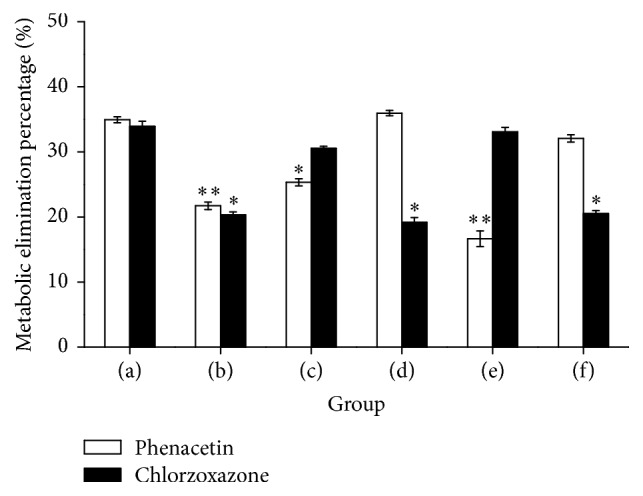
Metabolic elimination percentage of two probe substrates, phenacetin (for CYP1A2) and chlorzoxazone (for CYP2E1). (a) Control group; (b) PMR group; (c) CYP1A2 inhibitor control group; (d) CYP2E1 inhibitor control group; (e) CYP1A2 inhibitor and PMR group; (f) CYP2E1 inhibitor and PMR group (shown as mean SD, *n* = 5; ^*∗*^significant difference compared with the control group, *P* < 0.05; ^*∗∗*^extremely significant difference compared with the control group, *P* < 0.01).

The activities of CYP1A2 and CYP2E1 in the rat liver from all groups were assayed using percentage of metabolic elimination of two probe substrates, phenacetin and chlorzoxazone, respectively (Sections 2.6 and 2.9). Compared with the control group, the activity of CYP1A2 enzyme was significantly decreased in PMR group (*P* < 0.01), CYP1A2 inhibitor control group (*P* < 0.05), and CYP1A2 + PMR inhibitor group (*P* < 0.01), whereas the activity of CYP2E1 enzyme was significantly decreased in PMR group (*P* < 0.05), CYP2E1 inhibitor group (*P* < 0.05), and CYP2E1 + PMR inhibitor group (*P* < 0.05) (Figure 6). The results demonstrated that oral administration of PMR could reduce activities of both CYP1A2 and CYP2E1, while CYP1A2 or CYP2E1 inhibitors had specific inhibition to CYP1A2 or CYP2E1 activities, respectively, in rat liver. CYP1A2 and CYP2E1 enzyme activities were significantly inhibited in CYP1A2 inhibitor + PMR group and CYP2E1 inhibitor + PMR group, respectively.

## 4. Discussions

The current work showed that oral administration of the aqueous extract of PMR did not affect rat serum ALT or AST levels significantly even at dosage of 40 g/kg BW that is 400-fold the clinical dosage according to the current Chinese Pharmacopoeia. This is consistent with our earlier report [34]. However, rats treated with liver cytochrome P450 enzyme CYP1A2 or CYP2E1 inhibitor with the same dosage of aqueous extract of PMR showed significantly elevated serum transaminase levels. Liver histologic observation revealed that the hepatocytes of experimental groups (CYP1A2 inhibitor + PMR or CYP2E1 inhibitor + PMR) showed cytoplasmic hydropic change (CYP2E1 inhibited) or hepatocellular karyopyknosis, which is an early stage of apoptosis (CYP1A2 inhibited).

The very important phenomenon is that serum transaminases ALT and AST levels increased more significantly during repeated PMR treatment after 2 interval times. Rat serum ALT and AST levels significantly increased in the group with the administration of PMR and CYP1A2 or CYP2E1 inhibitor compared with all control groups at 1 d, 3 d, 5 d, 7 d, and 22 d, but no greater than twofold, which suggested that PMR related hepatotoxicity that happened in clinic might be due to some people with lower activities of CYP1A2 or CYP2E1 from genetic polymorphism [22, 23]. Actually, there was a clinical report which suggested that PMR induced hepatotoxicity may relate to low activity of CYP1A2 [28]. ALT and AST levels increased nearly twofold in experimental groups (CYP1A2 inhibitor + PMR or CYP2E1 inhibitor + PMR) compared with all control groups at 44 d in accordance with the features of PMR related liver injury in the clinic which showed that more severe liver damage happened when PMR was taken repeatedly. Rat serum ALT and AST levels were decreasing at 46 d compared to at 44 d, suggesting that drug resistance may happen. In order to eliminate false-positive results from different groups, ALT and AST levels among all groups in the interval days (at 37 d, in the middle of 3-week interval days) were tested and no significant group difference in all groups was found, which is also in accordance with the features of PMR related hepatotoxicity that happened in the clinic where liver injury can subside after drug withdrawal.

PMR has been used widely as a medicine in the clinic or as nutritional supplements in China and some other countries. Hundreds of acute hepatitis cases related to PMR have been reported from all over the world [35]. According to the relevant research, the main type of liver injury caused by PMR was hepatocellular. One of the clinical studies reported that the main type of liver injury caused by PMR was hepatocellular (77.8%), cholestatic (5.6%), and mixed (16.7%) [11]. Histological findings exhibited fatty change, infiltration of neutrophils, even apoptotic body, bridging necrosis, and fibrosis in the liver [36], consistent with acute hepatitis laboratory data in which ALT and AST levels elevate remarkably in almost all patients, and total bilirubin and alkaline phosphatase elevate in some patients [33]. In our study, rats in groups of CYP1A2 inhibitor + PMR or CYP2E1 inhibitor + PMR were found to show hepatotoxicity such as hepatocellular hydropic change or karyopyknosis, sinusoid moderate expansion, and congestion in the portal tract, plus significantly elevated ALT and AST levels, especially after repeated administration of PMR. Obviously, the histological feature and biochemical data were not completely consistent with clinical data, indicating that low activity of CYP1A2 or CYP2E1 is just one of the important reasons for PMR related liver injury. Further efforts are needed to reveal other influence factors and declare the mechanisms of PMR related hepatotoxicity.

The possible relationship between inhibition of CYPs and the PMR induced hepatotoxicity suggests that metabolism of some components in the aqueous extract of PMR was retarded. Stilbenes, anthraquinones, and lecithin are known to be the main active substances in PMR [37]. HPLC analysis showed that there were four main well-separated chromatographic peaks standing for THSG, EG, emodin, and physcion in the aqueous extract of PMR (Figure 2). Emodin was found to be the main component of anthraquinones in PMR. Previously, we found that emodin in 95% ethanol-eluted extract of PMR exhibits cytotoxicity and causes cell S phase arrest and apoptosis in human liver L02 cells [38]. Similar results were observed by other research groups [39]. It has been shown that anthraquinones can be metabolized by CYPs, and emodin is known to be metabolized by CYP1A2 enzyme [40]. There were in vivo studies that suggested that emodin may induce rat hepatic lesions [41, 42]. But the main chemical constituents of PMR related to hepatotoxicity need further investigation.

The activity assay of CYPs showed that the activity of CYP2E1 or CYP1A2 was significantly inhibited by the aqueous extract of PMR, but CYP2E1 activity was obviously increased in group (e) (CYP1A2 inhibitor + PMR) or CYP1A2 activity was obviously increased in group (f) (CYP2E1 inhibitor + PMR) after prolonged and repeated administration of PMR. The possible explanation could be that the given CYP inhibitors may have nonspecific targets which may influence some undefined activities of CYP450 enzymes isoforms. Besides CYP1A2 and CYP2E1, the genetic polymorphism of other CYP450 enzymes might be related to the PMR induced hepatotoxicity as well, and the clinical PMR related hepatotoxicity may result from multiple genetic polymorphisms of CYP450 enzymes or some epigenetic modification. All the experimental results from rats need to be confirmed with clinical data, which requires us to collaborate closely with clinical doctors in the future.

## 5. Conclusions

In conclusion, we have shown that serum transaminases ALT and AST were increased significantly and moderate liver injury appeared in liver histopathological observation, after the administration of PMR in CYP1A2 or CYP2E1 inhibited rats; the result suggested that low level activity of CYP1A2 or CYP2E1 from genetic polymorphism among people might be one of the important reasons for the hepatotoxicity induced by PMR in clinical practice.

## Figures and Tables

**Table 1:** Contents of the main components in the aqueous extract of PMR.

Components	Content (*µ*g/mL)	Percentage of content (%)
THSG	49.04 ± 4.73	0.4100 ± 0.0400
EG	6.84 ± 0.16	0.0570 ± 0.0010
Emodin	0.91 ± 0.09	0.0080 ± 0.0008
Physcion	0.31 ± 0.01	0.0030 ± 0.0001
